# LISTENING TO COMMUNITY VOICES FOR COLLECTIVE ACTION: THE OBESITY BILL OF RIGHTS ORIGINS

**DOI:** 10.1093/geroni/igae098.1703

**Published:** 2024-12-31

**Authors:** Dorothea Vafiadis

**Affiliations:** National Council on Aging, Arlington, Virginia, United States

## Abstract

Four town hall meetings were convened by the National Council on Aging and National Consumers League in senior centers and faith-based organizations during the summer of 2023. These venues were in four different states representing diverse cultural communities and socioeconomic backgrounds. Over 250 older adults, community leaders, and clinicians participated in discussions on the complex social and institutional barriers to obtaining appropriate management of overweight/management. These discussions yielded themes that were translated into the 8 core principles of the Obesity Bill of Rights (OBOR) which address issues such as ageism, weight bias, and aspects of the healthcare system that preclude adequate obesity care, coverage, and reimbursement. Obesity experts and stakeholders reviewed and validated the OBOR in the fall of 2023. On January 31, 2024, the OBOR was officially launched as a framework for health equity and self-advocacy. To date, nearly 50 health organizations and medical societies have endorsed the OBOR.

